# A Case of Drug-Induced Lupus in Crohn’s Disease During Infliximab Therapy

**DOI:** 10.7759/cureus.74782

**Published:** 2024-11-29

**Authors:** Tomotaka Tanaka, Daiki Hirano, Syohei Ishimaru, Keiko Arataki

**Affiliations:** 1 Department of Gastroenterology, Tsuchiya General Hospital, Hiroshima, JPN

**Keywords:** antinuclear antibody, crohn disease, drug-induced lupus erythematosus, infliximab, vedolizumab

## Abstract

A 46-year-old man with a known case of Crohn's disease, which developed in August 2010, had been in remission since then with infliximab treatment. However, in November 2023, he developed photosensitivity, followed by joint pain and general fatigue in December. Blood tests revealed positive antinuclear antibodies, leading to a diagnosis of drug-induced lupus. After switching from infliximab to vedolizumab, his symptoms improved, and antinuclear antibodies became negative. We present a case of Crohn's disease in which drug-induced lupus developed after approximately 14 years of infliximab treatment. Cases of drug-induced lupus developing from long-term administration of tumor necrosis factor alpha (TNFα) inhibitors are rare, so we report this case with a literature review.

## Introduction

Infliximab (IFX), an anti-tumor necrosis factor alpha (anti-TNFα) inhibitor, is used for induction and maintenance therapy in Crohn's disease. IFX is also administered for various conditions, including inflammatory bowel diseases like Crohn's disease and ulcerative colitis, rheumatoid disorders, psoriasis, and ankylosing spondylitis. However, it is well-known that IFX can cause immune-mediated adverse effects, such as infections and an increased risk of malignant lymphoma.

We encountered a case of drug-induced lupus (DIL) suspected to be caused by IFX. The incidence of DIL due to anti-TNFα inhibitors is reported to be between 0.18% and 0.41% in Western countries, with an average onset period of 41 weeks [1]. However, reports of long-term use cases, such as this one with approximately 14 years of IFX administration, are rare. Therefore, we present this case with a brief literature review.

## Case presentation

The case involves a 46-year-old male presenting with chief complaints of joint pain throughout the body, general fatigue, and skin erythema. He has no past medical or family history, and he drinks socially on occasion. In August 2010, the patient presented with abdominal pain and fever. A colonoscopy revealed small and large bowel Crohn's disease. Initial treatment with oral 5-aminosalicylic acid (5-ASA) at 3000 mg did not improve symptoms, so induction therapy with IFX 300 mg was initiated. Maintenance therapy with IFX was continued every eight weeks, but the patient experienced recurrent anal fistulas in 2012 and 2020. After surgeries for these fistulas, IFX induction therapy was restarted, followed by maintenance therapy every eight weeks.

**Figure 1:**
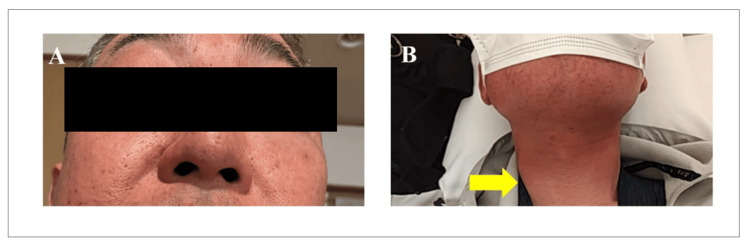
Photos of the face and skin (A, B) Exposed areas of the skin, such as the face and neck (yellow arrow indicates the borders), exhibit redness and erosion after exposure to ultraviolet light (photosensitivity).

**Figure 2:**
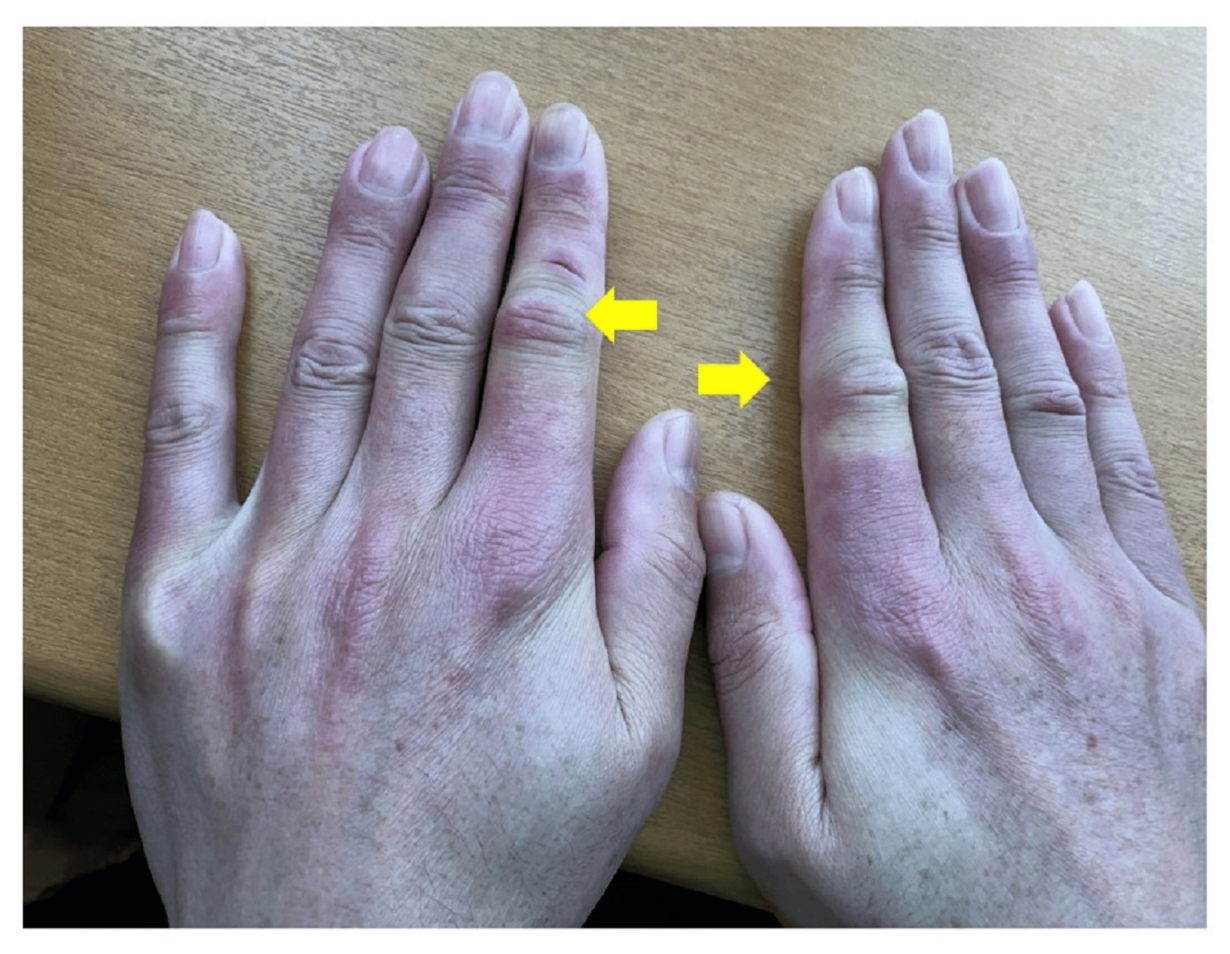
Photo of the two hands showing a mild swelling in the second joint of both fingers (indicated by the yellow arrows)

In November 2023, the patient developed prominent erythema on exposed skin areas, such as the face and neck (Figure 1), and was diagnosed with photosensitivity by a dermatologist. By December, he began experiencing generalized joint pain, particularly in the second joint of both hands (Figure 2), along with general fatigue.

Upon re-evaluation at our department, blood tests (Table 1) showed elevated antinuclear antibodies (ANA), anti-ssDNA IgM antibodies, and anti-dsDNA IgM antibodies, raising suspicion of DIL.

**Table 1:** Blood tests

Parameters	Results
White blood cells	4140/μl
Neutrophils	51.7%
Lymphocytes	37.7%
Monocytes	8.9%
Eosinocytes	1.2%
Basophils	0.5%
Red blood cells	481 × 104/μl
Hemoglobin	15.6 g/dl
Hematocrit	46.50%
Platelets	22.6 × 104/μl
Total protein	7.6 g/dl
Albumin	4.3 g/dl
Aspartate aminotransferase	28 U/l
Alanine aminotransferase	41 U/l
Cholinesterase	332 U/l
Lactate dehydrogenase	193 U/l
Total bilirubin	0.5 mg/dl
Blood urea nitrogen	12 mg/dl
Creatinine	0.77 mg/dl
Na	139.1 mEq/l
K	4.6 mEq/l
Cl	102 mEq/l
C-reactive protein	0.02 mg/dl
Rheumatoid factor	0.0 IU/ml
IgG	1516 mg/dl
IgA	412 mg/dl
IgM	256 mg/dl
Complement C3	109 mg/dl
Complement C4	21.3 mg/dl
Total hemolytic complement activity	46.9 CH50/mL
Antinuclear antibodies	× 160
Anti-DNA antibodies	4 IU/ml
Anti-double-stranded-DNA antibodies IgG	1.1 IU/ml
Anti-double-stranded-DNA antibodies IgM	10 U/ml
Anti-ss-DNA antibodies IgG	3.3 IU/ml
Anti-ss-DNA antibodies IgM	101 IU/ml
Anti-histone antibodies	<1.0

**Figure 3:**
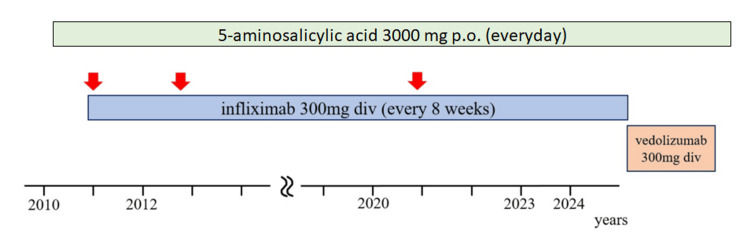
Clinical course

Consequently, IFX was replaced with vedolizumab (VED). Within two months, ANA levels normalized, and by three months, the joint pain and skin symptoms had improved. Vedolizumab is now being administered every eight weeks, and there has been no recurrence of DIL reported to date (Figure 3).

## Discussion

As biological agents are increasingly used for a variety of diseases, unexpected adverse events have become more common. One such adverse event is DIL caused by anti-TNFα inhibitors. DIL is characterized by clinical symptoms and immunological abnormalities similar to those of systemic lupus erythematosus (SLE), triggered by various drugs [2]. Traditionally, hydralazine, procainamide, and isoniazid have been commonly reported as causes, referred to as classical DIL [3]. In recent years, reports of DIL induced by anti-TNFα inhibitors and chemotherapeutic agents have increased. A notable feature of DIL is its improvement upon discontinuation or replacement of the causative drug [2].

The clinical manifestations of DIL are diverse and generally milder compared to idiopathic SLE. Common symptoms include fever, general fatigue, joint pain, and serositis, while renal involvement, central nervous system symptoms, hematological abnormalities, and rashes are rare. Almost all DIL cases present with positive ANA. There are slight differences in clinical features between classical DIL and anti-TNFα inhibitor-induced DIL. Both types commonly present with fever, joint pain, and myalgia, but rashes are more frequent in anti-TNFα inhibitor-induced DIL, whereas serositis is more frequent in classical DIL. Renal involvement and central nervous system symptoms are rare in both types, but severe clinical presentations such as glomerulonephritis can occur in anti-TNFα inhibitor-induced DIL. Laboratory differences include a higher frequency of leukopenia, hypocomplementemia, and anti-dsDNA antibody positivity in anti-TNFα inhibitor-induced DIL, whereas classical DIL has a higher frequency of anti-histone antibody positivity [4-6].

The diagnostic criteria for DIL are not yet established, often following the American College of Rheumatology's criteria for SLE [7]. Borchers et al. proposed the following criteria for suspected DIL: (1) sufficient exposure to a specific drug for a defined period, (2) presence of at least one symptom fitting SLE, (3) absence of SLE history before the suspect drug's administration, and (4) symptom improvement within weeks after discontinuing the suspect drug. Our case met all these criteria, strongly suggesting DIL [8].

Type I interferons (IFN-α/β) play a central role in SLE pathogenesis. Apoptotic cells release nucleic acids that form immune complexes with autoantibodies in SLE patients, stimulating plasmacytoid dendritic cells (pDC) to produce IFN-α [9]. The mechanism by which anti-TNFα inhibitors induce SLE is not fully understood, but in vivo studies have shown that TNFα suppresses the production of pDC and IFN-α from hematopoietic progenitor cells. Neutralizing TNFα restores IFN-α production from pDC, suggesting a cross-regulatory relationship between TNFα and IFN-α [10]. Thus, increased IFN-α levels due to anti-TNFα inhibitors may trigger SLE. Other hypotheses include the release of autoantigens from apoptotic cells binding to cell surface-expressed TNF, suppression of Th1 responses with a shift toward Th2 responses, and enhanced autoantibody production due to B lymphocyte activation from increased infections associated with anti-TNFα inhibitor use [11-13].

Reports of DIL associated with anti-TNFα inhibitors are most common in rheumatoid arthritis patients, but cases have also been reported in Crohn's disease, ulcerative colitis, psoriatic arthritis, and ankylosing spondylitis, indicating no disease-specific predisposition [14]. DIL has been reported with not only IFX but also etanercept (ETC) and adalimumab (ADA). The BIOGEAS project in Spain reported 140 cases of DIL induced by anti-TNFα inhibitors up to July 2009, with an incidence of 37% for IFX, 33% for ETC, and 25% for ADA [15]. These DIL cases frequently exhibited skin symptoms and high rates of anti-dsDNA antibody positivity, consistent with previous reports. Interestingly, 63.8% of rheumatoid arthritis patients and 49.1% of Crohn's disease patients treated with IFX developed ANA positivity, with anti-dsDNA antibody positivity rates of 13% and 21.5%, respectively [16]. Beigel et al. reported that anti-dsDNA antibody positivity, rather than increased ANA titers, was associated with DIL [17]. They also noted that higher age at anti-TNFα inhibitor initiation and concomitant immunomodulator use might protect against DIL onset.

DIL occurrence often necessitates discontinuing the causative drug, but switching to a different anti-TNFα inhibitor without adverse effects has been reported [18]. Subramanian et al. found that among eight inflammatory bowel disease patients who developed DIL from IFX, only two experienced symptom recurrence after switching to another anti-TNFα inhibitor [19]. In our case, DIL improved upon switching from IFX to VED, but improvement might also have been possible with a different anti-TNFα inhibitor.

As the number of inflammatory bowel disease patients increases, the use of anti-TNFα inhibitors is also expected to rise, likely leading to more encounters with adverse effects. Although DIL is a rare adverse effect of anti-TNFα inhibitors, early recognition and appropriate management are crucial.

## Conclusions

We presented a case of DIL following long-term IFX therapy in a patient with Crohn's disease. To our knowledge, there are no previous reports of DIL developing after long-term administration of IFX (for 14 years), as observed in this case. The condition improved promptly after switching medications. This case highlights the importance of considering the potential for DIL when managing patients undergoing IFX therapy.
